# Statement of Retraction

**DOI:** 10.1080/2090598X.2020.1832358

**Published:** 2020-10-28

We, the Editors and Publishers of the Arab Journal of Urology, upon the requests of the authors have retracted the following article:

Mohamed A. Fouda, Ahmed A. Shokeir, Ehab W. Wafa, Ayman F. Refaie, Tarek El Diasty, Mona Abdelrahim, Mohamed A. Sobh & Mohamed A. Ghoneim (2011) Hyperechogenic renal parenchyma in potential live related kidney donors: Does it justify exclusion?, Arab Journal of Urology, 9:4, 235-239, http://doi.org/10.1016/j.aju.2011.10.013

We are now cognizant of a substantially similar version of this article which was previously submitted to, and published in, the *African Journal of Nephrology*, as https://doi.org/10.21804/2-1-857

We have been informed in our decision-making by our policy on publishing ethics and integrity and the COPE guidelines on retractions.

The retracted article will remain online to maintain the scholarly record, but it will be digitally watermarked on each page as “Retracted.”

